# Central Nervous System Involvement and Neuroradiological Imaging Insights of Neurocutaneous Melanocytosis in Congenital Melanocytic Nevi

**DOI:** 10.3390/diagnostics14212345

**Published:** 2024-10-22

**Authors:** Christer Ruff, Georg Gohla, Thomas Nägele, Marion Batra

**Affiliations:** Department of Diagnostic and Interventional Neuroradiology, Eberhard Karls-University Tübingen, Hoppe-Seyler-Str. 3, 72076 Tübingen, Germany

**Keywords:** central nervous system (CNS), congenital melanocytic nevi (CMN), giant congenital melanocytic nevi (GCMN), magnetic resonance imaging (MRI), neurocutaneous melanocytosis (NCM)

## Abstract

Congenital melanocytic nevi (CMN) are pigmented lesions present at birth, varying widely in size and clinical impact. In rare instances, these nevi become visible during the first months of life, a phenomenon known as tardive melanocytic nevi (tardive CMN). Giant congenital melanocytic nevi (GCMN) are defined as nevi larger than 40 cm in projected adult size (PAS). Their association with the central nervous system (CNS) poses significant risks, including melanoma and neurocutaneous melanocytosis (NCM), where melanocytes infiltrate the CNS, potentially causing seizures, hydrocephalus, and, rarely, CNS melanoma. MRI is recommended for GCMN patients, particularly those with numerous satellite nevi or neurological symptoms, to detect CNS involvement. The Nevosurgery Network recommends MRI examinations in cases of GCMN (>40 cm PAS), the presence of over 20 concomitant nevi, and neurological symptoms requiring clarification. CMN can be associated with melanocyte accumulations and melanin deposits in the brain, spinal cord, and leptomeninges.

Congenital melanocytic nevi (CMN) show considerable variability in color, color irregularity, surface, texture, presence of superficial or deep nodules, and extent of hypertrichosis. Studies have been unable to demonstrate any prognostic relevance of these morphological features for the risk of melanoma [1]. Therefore, the most important prognostic parameters are the size and number of accompanying nevi. Concerning localization, head and extremity nevi have a lower tendency to degenerate than trunk nevi [2,3]. A common classification system distinguishes between four size categories for CMN, which are abbreviated as SCMN (small, <1.5 cm), MCMN (medium, 1.5–20 cm), LCMN (large, >20–40 cm), and GCMN (giant, >40 cm), in relation to the expected size at adulthood, which is calculated using the projected adult size (PAS) formula [4].

CMN with similar diameters can exhibit significant differences in other characteristics. In particular, the number of accompanying nevi is a considerable factor. The presence of more than 20 accompanying nevi indicates an elevated risk of central nervous system (CNS) involvement [5]. The combination of cutaneous and CNS manifestations is called neurocutaneous melanocytosis or neurocutaneous melanosis (NCM). If two or more CMN are present, an MRI is recommended if their diameter exceeds 1.5 cm [6,7]. The Nevosurgery Network, therefore, recommends MRI examinations in cases of GCMN (>40 cm projected adult size (PAS)), and/or the presence of >20 concomitant nevi, and/or neurological symptoms requiring clarification [8]. In addition, there is a rare clinical phenotype in which children do not have a distinctly large main nevus but rather multiple, usually three to ten, medium-sized CMN. This phenotype is associated with an exceptionally high risk of symptomatic CNS involvement. It is referred to as “multiple medium-sized MCMN” (mMCMN) [4,9,10]. An MRI should be performed on these children in all cases.

For illustrative purposes, the CNS findings of the two presented 11- and 23-day-old female patients (Figure 1) with a GCMN and over 50 accompanying nevi are depicted in Figure 2 and Figure 3 for the first patient and Figure 4 for the second patient, respectively. MRI acquisition parameters are shown in Table 1. When the CNS is involved, it is crucial to differentiate between focal melanosis of the brain parenchyma, which is more prevalent, and disseminated or diffuse melanosis of the leptomeninges. While focal melanosis is more likely to precipitate epileptic seizures, diffuse melanosis is significantly more frequently associated with complications such as hydrocephalus or even cerebral melanoma formation [7]. At the age of the two presented patients, neural melanosis is not obscured by myelination and can be visualized without contrast. As the brain matures (myelination, size) over the middle and later months of the first year, the melanosis becomes more difficult to identify and additional contrast-enhanced T1-weighted sequences might be necessary [11]. In view of the literature, as in our two patients, there are manifestations, in particular, of the temporal lobe, cerebellum, pons, and/or medulla, and possibly also an increased incidence of arachnoid cysts [12].

With evidence of CNS involvement on MRI, the localization of the melanin is crucial. Developmental abnormalities and indications for neurosurgical intervention are significantly more prevalent in patients with both cutaneous and leptomeningeal seeding of melanin on MRI in contrast to patients with focal intraparenchymal melanin localization [7]. Current studies with limited follow-up time show that the rate of melanoma is higher with positive MRI (detection of melanin in the CNS) than in patients with negative MRI (12% vs. 2%) [17].

A common query concerns the necessity of additional follow-up MRIs. There is a lack of consensus on specific further screening and recommendations [14]. Even if an initial MRI shows an abnormality, an imaging follow-up should typically only be performed if there is a change in the clinical findings, e.g., neurological symptoms. The management of CMN has shifted towards individualized care, emphasizing the balance between malignancy risk and the psychosocial impact of visible lesions. Surgical treatment, often performed for cosmetic reasons, is now approached more conservatively, aiming to minimize surgical burden while improving cosmetic outcomes. A multidisciplinary approach involving dermatological, surgical, neurological, and psychological care is essential for addressing the complex needs of CMN patients [1,8].

## Figures and Tables

**Figure 1:**
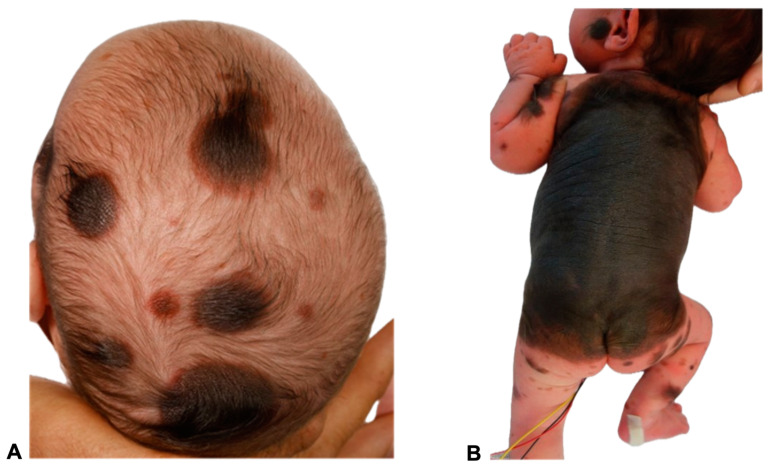
Images of two 11- and 23-day-old female patients with congenital melanocytic nevi (CMN) showing multiple small- to medium-sized nevi (>50) all over the body, including the head (**A**,**B**) and extremities (**B**). A giant congenital melanocytic nevi (GCMN) extends over the back and encircles the torso (**B**). The clinical term CMN encompasses a range of melanocytic spots and plaques with varying characteristics. Any skin location may be affected. In many children, CMN manifest as isolated skin moles, whereas in others, they are accompanied by numerous satellite nevi, sometimes referred to as so-called accompanying nevi. CMN are typically distinguished by the presence of nevomelanocytes, or nevus cells, which form well-ordered clusters in the epidermis and organize into cords, sheets, or nests within the dermis. In contrast to acquired nevi, CMN are more likely to extend deeper into the dermis or even into the subcutaneous fat layer [11]. These nevus cells frequently congregate in the vicinity of dermal appendages, including hair follicles, neurovascular structures, and sebaceous glands [11,12]. Additionally, they may extend as cords of cells between collagen bundles in the reticular dermis [13]. Besides showing deep dermal involvement in the neonatal period, the nevus cell depth is not modified with age [14,15]. Concerning genetics, CMN of all sizes are most commonly caused by mutations in the NRAS gene. Mutations in BRAF are a much rarer cause of multiple CMN and are frequently associated with a multinodular phenotype. In a patient cohort presented by Polubothu et al., there was no correlation between genotype and differences in the incidence of neurological disease during childhood [16].

**Figure 2:**
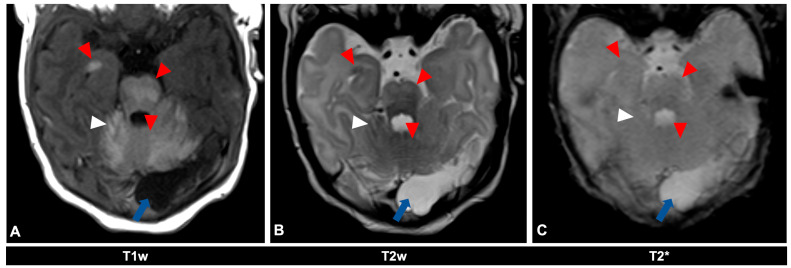
MR imaging of an 11-day-old female patient with neurocutaneous melanosis (NCM) using an MRI-compatible incubator. (**A**–**C**) Imaging findings of leptomeningeal (white arrowhead) and intraparenchymal melanosis (red arrowhead) demonstrate a high T1 signal, especially in the pons, cerebellum, and amygdala. These findings are not sufficiently detectable in T2-weighted (**B**) or T2 star (**C**) sequences (white arrowhead), at most a slight signal reduction in T2-weighted sequences. The patient also has an infratentorial arachnoid cyst (blue arrow). Spinal MRI showed no abnormalities (not shown). T1w = T1-weighted; T2w = T2-weighted; T2* = T2 star.

**Figure 3:**
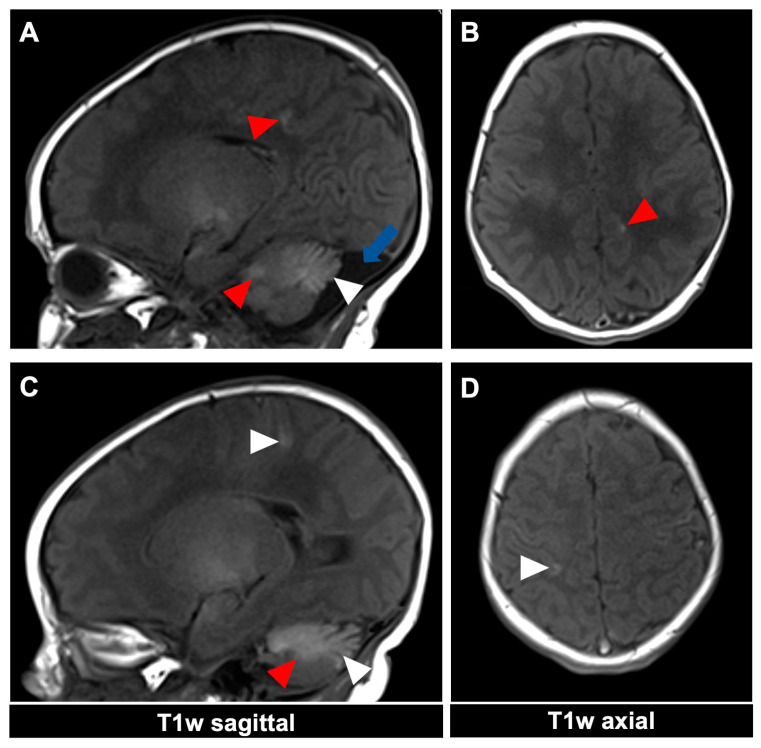
MR imaging of an 11-day-old female patient with neurocutaneous melanosis (NCM) using an MRI-compatible incubator. (**A**–**D**) Imaging findings of leptomeningeal (white arrowhead) and intraparenchymal melanosis (red arrowhead) demonstrate a high T1 signal in sagittal and corresponding axial planes. The image examples illustrate that acquiring two different planes of T1-weighted sequences is advantageous, as it is sometimes difficult to distinguish between leptomeningeal and intraparenchymal manifestations. The patient also has an infratentorial arachnoid cyst ((**A**), blue arrow). T1w = T1-weighted.

**Figure 4:**
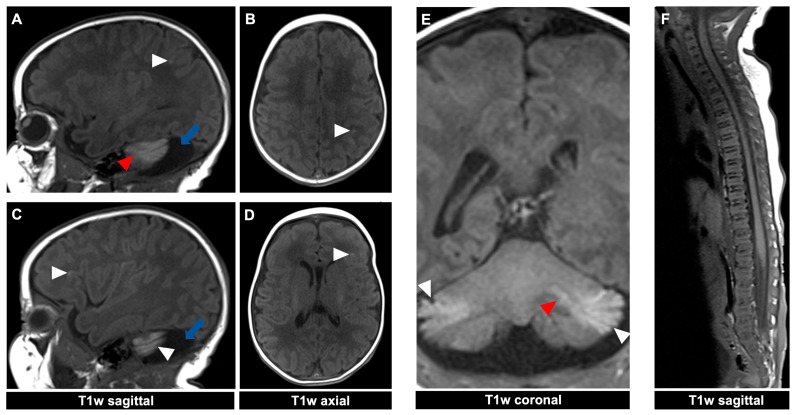
MR imaging of a 23-day-old female patient with neurocutaneous melanosis (NCM) using an MRI-compatible incubator. (**A**–**E**) Intraparenchymal (red arrowhead) and leptomeningeal (white arrowhead) melanosis imaging findings demonstrate a high T1 signal. The acquired sagittal and corresponding axial image examples (**A**–**D**) illustrate that acquiring two different planes of T1-weighted sequences is advantageous, as it is sometimes difficult to distinguish between leptomeningeal and intraparenchymal manifestations. The patient also has an infratentorial arachnoid cyst ((**A**,**C**) blue arrow). (**F**) Spinal MRI showed no abnormalities. T1w = T1-weighted.

**Table 1:** MRI acquisition parameters using the 1.5 T scanner Magnetom Aera (Siemens Healthineers, Erlangen, Germany).

Parameters	Brain					Spine
	T1 SE Axial	T1 SE Sagittal	T1 SE Coronal	T2 TSE Axial	T2* Axial	T1 TSE Sagittal
Field of view (mm)	160	160	160	160	200	260
Voxel size (mm)	0.6 × 0.6 × 3.0	0.6 × 0.6 × 3.0	0.6 × 0.6 × 3.0	0.6 × 0.6 × 3.0	0.4 × 0.4 × 4.0	0.3 × 0.3 × 2.0
Slice thickness (mm)	3	3	3	3	4	2
Number of slices	32	24	26	32	22	16
Base resolution	256	256	256	256	256	432
TR (ms)	610	510	552	6600	828	400
TE (ms)	11	12	12	104	24.6	12
Averages	3	2	2	5	3	5
Concatenations	1	1	1	1	1	2
Flip angle	60	60	60	126	20	112

SE = Spin Echo; TSE = Turbo Spin Echo; T2* = T2 star; TR = repetition time; TE = echo time.

## Data Availability

All relevant data are contained within the article.

## References

[1] Mologousis M.A., Tsai S.Y.C., Tissera K.A., Levin Y.S., Hawryluk E.B. (2024). Updates in the Management of Congenital Melanocytic Nevi. Children.

[2] Bett B.J. (2005). Large or multiple congenital melanocytic nevi: Occurrence of cutaneous melanoma in 1008 persons. J. Am. Acad. Dermatol..

[3] DeDavid M., Orlow S.J., Provost N., Marghoob A.A., Rao B.K., Huang C.L., Wasti Q., Kopf A.W., Bart R.S. (1997). A study of large congenital melanocytic nevi and associated malignant melanomas: Review of cases in the New York University Registry and the world literature. J. Am. Acad. Dermatol..

[4] Krengel S., Scope A., Dusza S.W., Vonthein R., Marghoob A.A. (2013). New recommendations for the categorization of cutaneous features of congenital melanocytic nevi. J. Am. Acad. Dermatol..

[5] Marghoob A.A., Dusza S., Oliveria S., Halpern A.C. (2004). Number of satellite nevi as a correlate for neurocutaneous melanocytosis in patients with large congenital melanocytic nevi. Arch. Dermatol..

[6] Neale H., Plumptre I., Belazarian L., Wiss K., Hawryluk E.B. (2022). Central nervous system magnetic resonance imaging abnormalities and neurologic outcomes in pediatric patients with congenital nevi: A 10-year multi-institutional retrospective study. J. Am. Acad. Dermatol..

[7] Waelchli R., Aylett S.E., Atherton D., Thompson D.J., Chong W.K., Kinsler V.A. (2015). Classification of neurological abnormalities in children with congenital melanocytic naevus syndrome identifies magnetic resonance imaging as the best predictor of clinical outcome. Br. J. Dermatol..

[8] Ott H., Krengel S., Beck O., Böhler K., Böttcher-Haberzeth S., Cangir Ö., Fattouh M., Häberle B., Hüging M., Königs I. (2019). Multidisciplinary long-term care and modern surgical treatment of congenital melanocytic nevi—Recommendations by the CMN surgery network. J. Dtsch. Dermatol. Ges..

[9] Neuhold J.C., Friesenhahn J., Gerdes N., Krengel S. (2015). Case reports of fatal or metastasizing melanoma in children and adolescents: A systematic analysis of the literature. Pediatr. Dermatol..

[10] Price H.N., Schaffer J.V. (2010). Congenital melanocytic nevi-when to worry and how to treat: Facts and controversies. Clin. Dermatol..

[11] Barnhill R.L., Fleischli M. (1995). Histologic features of congenital melanocytic nevi in infants 1 year of age or younger. J. Am. Acad. Dermatol..

[12] Escandon-Perez S., Landeta-Sa A.P., González-Jasso Y., Arenas-Guzmán R. (2019). Giant congenital melanocytic nevi. Bol. Med. Hosp. Infant. Mex..

[13] Arneja J.S., Gosain A.K. (2009). Giant congenital melanocytic nevi. Plast. Reconstr. Surg..

[14] Ruiz-Maldonado R., Tamayo L., Laterza A.M., Durán C. (1992). Giant pigmented nevi: Clinical, histopathologic, and therapeutic considerations. J. Pediatr..

[15] Fenton D.A., Mayou B., Atherton D., Black M.M. (1987). Histopathology of Giant Congenital Melanocytic Nevi—Implications for Treatment. Br. J. Dermatol..

[16] Polubothu S., McGuire N., Al-Olabi L., Baird W., Bulstrode N., Chalker J., Josifova D., Lomas D., O’Hara J., Ong J. (2020). Does the gene matter? Genotype-phenotype and genotype-outcome associations in congenital melanocytic naevi. Br. J. Dermatol..

[17] Kinsler V.A., O’Hare P., Bulstrode N., Calonje J.E., Chong W.K., Hargrave D., Jacques T., Lomas D., Sebire N.J., Slater O. (2017). Melanoma in congenital melanocytic naevi. Br. J. Dermatol..

